# A Lightweight SPI-Flash Controller Based on AMBA AHB-Lite Bus

**DOI:** 10.3390/mi15101246

**Published:** 2024-10-10

**Authors:** Bingzheng Li, Weike Wang, Hanbing Chu, Zixuan Liu, Wei Wang

**Affiliations:** College of Electronic and Information Engineering, Shandong University of Science and Technology, Qingdao 266590, China; bingzhengli@sdust.edu.cn (B.L.); hanbingchu@sdust.edu.cn (H.C.); liuzixuan@sdust.edu.cn (Z.L.); wang_wei@sdust.edu.cn (W.W.)

**Keywords:** SPI-Flash controller, AMBA AHB-lite bus, lightweight, FPGA verification

## Abstract

The utilization of SPI-Flash in embedded systems is widespread, primarily serving as program storage during the boot process. As a result, the boot process is influenced to some extent by the SPI-Flash controller. This paper presents a lightweight SPI-Flash controller that simplifies the boot process design by establishing a direct connection between the SPI-Flash and AHB-lite bus interface, enabling rapid program execution in RAM instead of directly from the SPI-Flash. Additionally, the controller can function as a bare-metal program downloader for testing the boot process functionality during FPGA-based SoC (system-on-chip) prototype verification. The system-level simulation and FPGA verification results demonstrate that the proposed SPI-Flash controller successfully achieves its intended functional impact in operations to target the Micron N25Q256A SPI-Flash chip, boot process design, and bare-metal program download. The synthesis results under the SMIC 180 nm 1P8M technology process indicate that this SPI-Flash controller exhibits remarkable performance, power consumption, and area utilization. The source code of the proposed lightweight SPI-Flash controller has been uploaded to GitHub as an open-source project.

## 1. Introduction

SPI-Flash is a widely used type of non-volatile memory in embedded systems, primarily serving as the storage medium for user programs during the boot process. In the rapidly advancing landscape of electronic devices, memory controllers play a pivotal role in managing the storage and retrieval of data [1]. The SPI-Flash controller assumes responsibility for converting read requests from the bus into SPI timing signals for SPI-Flash control and transforming serial data from SPI-Flash into parallel data that aligns with the bus width. Ultimately, it transfers this parallel data to the system via the bus. Therefore, the program execution is influenced to some extent by the design of the SPI-Flash controller. The discussion in the related work section indicates that the program execution efficiency of embedded systems with an SPI-Flash controller mounted onto the APB bus is commonly limited by the performance of SPI-Flash and its controller, as it operates in the SPI-Flash XIP (execution in place) mode throughout the entire process. Mounting the SPI-Flash controller onto the AHB-lite bus offers more advantages for designing a boot process supporting RAM-based program execution, which eliminates these drawbacks associated with the XIP mode while also streamlining the design of the SPI-Flash controller.

### 1.1. Related Work

RISC-V processors have gained widespread adoption and experienced rapid development in recent years due to its lightweight design, high efficiency, and ease of customization. The porting of an open-source SoC based on a RISC-V processor to FPGA has emerged as a crucial approach for conducting hardware/software functional simulation tests in academic research. However, the verification process for some RISC-V SoC prototypes often focuses solely on user program execution while neglecting the crucial startup process of loading user programs from the SPI-Flash memory. Consequently, these RISC-V SoC prototypes frequently lack SPI-Flash controllers or employ different methods for loading user programs, leading to varying effects on program execution efficiency. A comparative analysis was conducted among the four open-source RISC-V SoC projects available on GitHub, namely HBird2 E203 [2], E906-SmartRun-Platform [3], zqh_riscv [4], and Yuheng [5]. The comparison focused on the interconnection between the SPI-Flash controller and CPU, as well as the program execution mode. Additionally, this study examines the various methodologies employed in the existing literature for SPI-Flash controller implementation.

The SPI-Flash controllers in HBirdv2 E203 and zqh_riscv are connected to the peripheral bus, whereas Yuheng’s SPI-Flash controller is directly linked to the processor via the instruction bus. The implementation of a more direct connection is expected to minimize uncertain factors and optimize the instruction fetching speed by shortening the instruction path from the SPI-Flash to the processor.

The entire program execution process in the three SoCs mentioned above is based on the XIP (execution in place) mode, where instructions are always sourced from SPI-Flash. This results in a heavy reliance on SPI-Flash performance, increased power consumption, and added complexity to both the SPI-Flash controller and SoC boot process design. Although there is no SPI-Flash controller implemented in the E906-SmartRun-Platform project, functional verification is achieved by directly initializing the binary executable of the user program into RAM before simulation or porting it to FPGA. This approach of fetching instructions proves more efficient and rational, as it leverages the faster read speed of RAM compared to SPI-Flash. In this scenario, only a dedicated SPI-Flash controller responsible for copying user programs to RAM needs to be developed, requiring only the execution of a fundamental read operation, thus reducing reliance on SPI-Flash during program execution and mitigating its impact on efficiency.

Therefore, the benefits and necessities of implementing a lightweight SPI-Flash controller based on the AHB-lite bus can be summarized as follows:

1. The AMBA bus, including the AHB-lite protocol, enjoys widespread acceptance both domestically and internationally and is the industry standard for on-chip bus architecture. Hence, the creation of communication protocols using the AMBA bus has significant current and future utility. SPI is a communication protocol that is frequently used in the AMBA bus architecture and has a variety of benefits, including user-friendliness, resource conservation, dependability, and stability [6]. Thus, the universality of an SPI-Flash controller based on an AHB-lite bus is enhanced.
2. The AHB2APB bridge is bypassed when compared to mounting the SPI-Flash controller onto the APB bus, resulting in a shortened path length for accessing the SPI-Flash controller. This leads to an improved upper limit for CPU access to SPI-Flash and reduces the risk during startup.
3. The SPI-Flash and IRAM (instruction random access memory) share the same instruction bus, which facilitates the implementation of arbitration between the SPI-Flash and IRAM during the process of copying user programs, thereby simplifying the design of the arbiter and the bootloader program.
4. The lightweight design of the SPI-Flash controller not only minimizes the area and power consumption of the embedded system but also enhances the portability of the SPI-Flash controller and simplifies user operations.

The implementation of a lightweight SPI-Flash controller based on the AHB-lite bus, however, will encounter challenges such as protocol conversion between AHB-lite and SPI protocols, power consumption optimization, and area reduction while ensuring the integrity of basic function implementation. Additionally, it should provide support for program downloading to SPI-Flash and loading programs into RAM for system initialization.

The design of our project is informed and inspired by several relevant studies conducted previously. The SPI-Flash controller consists of three layers, including the AHB slave interface, main control logic module, and ECC control module [7], as well as another SPI-Flash controller with three layers comprising the AHB slave interface, SPI-Flash command generator, and SPI command logic [8], serve as valuable references for designing the structure of the SPI-Flash controller in this paper. Two address spaces can be allocated for the SPI-Flash controller. One address space represents the SPI-Flash memory data array’s address space, which is accessed by an AHB-lite bus master for reading/writing data from/to the SPI-Flash memory array. The other address space is utilized to handle SPI commands through the SPI-Flash controller. These commands can be used as indices in a command configuration interpreter table that contains configuration information on command execution [9]. The transmission between the SPI-Flash controller and external SPI-Flash occurs serially, which means that instructions, addresses, and data cannot be sent simultaneously. Therefore, SPI-Flash access is completed in the form of a sequence by sending instructions first, followed by addresses, idle cycles, and finally, data transmission. In order to obtain the current state of SPI-Flash, the SPI-Flash controller autonomously reads SPI-Flash registers at regular intervals. When the value read from the register matches the expected value, a flag signal is generated to trigger the corresponding interrupt request. The external SPI-Flash is considered the internal memory of the system in the memory mapping mode. By configuring the relevant registers before accessing the SPI-Flash, the length of data can be determined by the control signal sent through the AHB bus. Consequently, the bus master can directly retrieve data from the SPI-Flash using its mapped address [10]. The interrupt mechanism, however, can only be activated once the configuration of the embedded system interrupt is completed during the boot phase. Until then, it is essential to implement mandatory measures to ensure that the CPU waits for the current operation to finish. Moreover, a reduction in periodic access to the SPI-Flash state register would result in saving more SPI timing and resources consumed by the SPI-Flash controller itself. It has been implemented by counter-based FSMs (finite state machines) in the proposed SPI-Flash controller. In the design of an open SPI-Flash controller [11], SPI-Flash operation commands are classified into different groups according to their common properties. One FSM is designed for one group rather than for each command to reduce the hardware resource usage. The concept of executing commands is further expanded by us, meaning that all commands are processed within the same FSMs. However, the parameters governing the state transition are adjusted based on the type of commands. Consequently, this approach leads to a more efficient utilization of hardware resources. One commonly used hot technique for communication based on the AHB-lite bus is to implement a decoder for fetching the address *HADDR* from the master. It decodes the HADDR to identify the slave selected for performing the transaction. When the slave is initially selected, it also monitors the status of *HREADY* to ensure that the previous bus transfer has been completed before it responds to the current transfer. A multiplexer embedded between the master and slave provides the purpose of multiplexing the fetched data from the slave (i.e., response) back to the master [12]. In this paper, the decoding and multiplexing functions are implemented by an arbiter, specially designed between the AHB-lite bus interface and the SPI-Flash controller.

The current SPI-Flash controller typically focuses on achieving high-speed communication with the SPI-Flash, which necessitates greater utilization of hardware resources and results in more pins, thereby increasing the power consumption and size of the embedded system. Considering the application requirements, such as loading programs from SPI-Flash to RAM during a system startup, it is possible to simplify the circuit design and realize a lightweight SPI-Flash controller while still meeting the rate requirements. Therefore, the objective of this paper is to design a lightweight SPI-Flash controller that can be directly mounted onto the AHB-lite bus. This controller not only facilitates basic SPI-Flash operations but also simplifies the boot process, enabling user program execution based on RAM instead of the XIP mode.

### 1.2. Contributions

The key contributions of our work encompass the following aspects:

1. A three-layer structure is adopted in the lightweight SPI-Flash controller. The SPI-Flash controller in this paper is designed with the following three-layer structure: AHB-lite slave interface, data transceiver, and serial–parallel converter. These layers facilitate the analysis of AHB-lite bus signals based on memory mapping, control of the SPI transmission, generation of SPI timing, and the response to the AHB-bus master.
2. The efficiency of SPI-Flash state determination has been enhanced through the replacement of periodic access to SPI-Flash state registers with an evaluation of the controller finite state machine’s status. Additionally, more stringent measures have been implemented to improve transmission stability when responding to the AHB-lite bus master.
3. The SPI-Flash controller proposed in this paper not only enables the implementation of the basic operations to SPI-Flash but also streamlines the boot process design for embedded systems. In addition, it can serve as a bare-metal program downloader, which is responsible for downloading bootloader programs to SPI-Flash.
4. In order to verify our design, the E906-SmartRun-Platform project is enhanced by addressing its absence of an SPI-Flash controller and boot process design. The corresponding RTL code, assemble code, and linking scripts can be accessed from the GitHub repository [13].

This paper is structured as follows: Section 2 presents the design details of the lightweight SPI-Flash controller, Section 3 shows the application of the SPI-Flash controller in designing the boot process for embedded systems and acting as a SPI-Flash downloader during FPGA-based SoC prototype verification, and Section 4 provides a comprehensive performance evaluation for the lightweight SPI-Flash controller. Finally, we conclude this paper in the last section.

## 2. The Design of the Lightweight SPI-Flash Controller

### 2.1. An Overview of the Lightweight SPI-Flash Controller Structure

**Figure 1:**
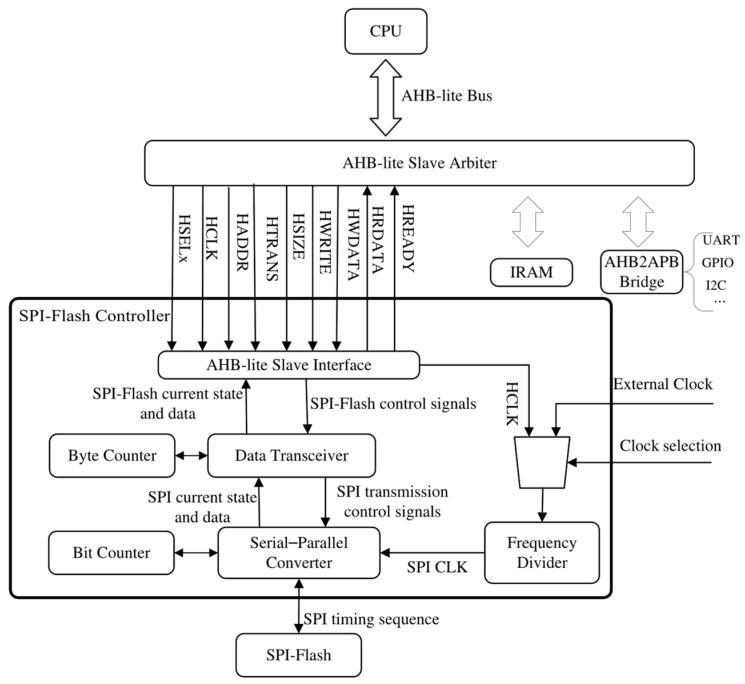
The structure of the proposed lightweight SPI-Flash controller.

The lightweight SPI-Flash controller, depicted in Figure 1, comprises three layers: an AHB-lite slave interface, a data transceiver, and serial–parallel converter.

Adhering to the principle of lightweight design, we have eliminated unnecessary functions and designs while optimizing command execution data processing and reducing data lines connecting with SPI-Flash. The *WriteEnable*, *SectorErase*, *Write*, and *Read* operations of SPI-Flash are implemented through an FSM, which saves design resources compared to implementing one FSM for each operation type. To ensure program copy stability and efficiency during startup, we canceled the indirect read and write modes as well as the FIFO buffer mechanism with high consumption. After data processing, serial data from SPI-Flash are transferred back to the bus with 32-bit granularity, while 32-bit data from the bus are written to SPI-Flash serially under the data transceiver’s control. The entire process follows counter principles where current SPI-Flash states can be judged by the stage of data processing, thereby avoiding access to the SPI-Flash state register, which saves clock cycles and related circuit design. The communication between the controller and SPI-Flash utilizes two MOSI/MISO lines, resulting in the reduced consumption of register configuration, I/O port usage, and power compared to QSPI without significantly affecting the transmission rates. The experimental results show that our proposed lightweight SPI-Flash controller provides sufficient throughput for small RAM-embedded systems’ startup requirements.

When asserted by the AHB-lite slave arbiter, the AHB-lite slave interface converts AHB-lite bus information regarding the SPI-Flash operations (including the *HWRITE*, *HADDR*, *HWDATA*, *HRDATA*, and *HSIZE* signals [14]) to the SPI-Flash control signals that indicate operation types, addresses, data, and size. Subsequently, based on these SPI-Flash control signals, commands, along with their corresponding addresses and data, are initialized to registers within the data transceiver. Each byte is then output to the serial–parallel converter according to the byte counter value. The *SPI CLK* during data transmission can be derived by dividing either the *HCLK* or the *external clock* with the frequency divider. Then, driven by the *SPI CLK*, the serial–parallel converter transfers each byte to SPI-Flash using the SPI protocol while ensuring successive reception of the data in bytes. By employing both the bit counter and byte counter mechanisms for control purposes, serial–parallel conversion and data concatenation are achieved following a principle that prioritizes transporting the most significant bits (MSB) within both the serial–parallel converter and data transceiver.

### 2.2. The Design of the AHB-Lite Slave Interface

The existence of the AHB-lite slave interface facilitates direct communication between the AHB-lite bus and the SPI-Flash controller. The AHB-lite slave interface not only analyzes the control information of the AHB-lite bus but also ensures that the SPI-Flash controller has sufficient time to complete corresponding operations by controlling the *HREADY* signal, which is utilized by the AHB-lite slave to indicate whether or not to initiate the next transfer.

#### 2.2.1. The Allocation of Space and Utilization of the SPI-Flash Controller

The SPI-Flash controller supports users in customizing the mapping of the *WriteEnable* and *SectorErase* functions, as well as memory space, to specific addresses during integration into an embedded system. This significantly enhances its adaptability to various embedded systems and SPI-Flash devices.

Initially, the base address of the SPI-Flash controller must be determined by referencing the existing peripheral’s address allocation within the current embedded system. Subsequently, the range for memory space can be established based on the size of the SPI-Flash. The *WriteEnable* and *SectorErase* functions can then be mapped to any address beyond the memory space range. For instance, if a base address of 0x7000_0000 is determined and a 256 KB SPI-Flash memory is expected to be controlled by the controller, then the offset for memory space with respect to the base address would span from 0x0000_0000 to 0x0004_0000. Additionally, functional addresses such as 0x0004_0008 and 0x0004_000C, which lie outside of this memory space range, can be assigned for the *WriteEnable* and *SectorErase* functions. Finally, an address space is allocated for the SPI-Flash controller, depicted in Table 1.

Based on the allocation of space, SPI-Flash can be easily manipulated: Writing any 32-bit data to the address (base address + 0x0004_0008) can enable writing or erasing operations. To execute the *SectorErase* function, the target erase sector’s address needs to be written to (base address + 0x0004_000C). Write operations can be achieved by directly writing the desired data to the address (base Address + offset) within the memory address corresponding to the physical address in SPI-Flash. Similarly, data in SPI-Flash can also be read directly from their corresponding addresses.

#### 2.2.2. The Finite State Machine Implemented in the AHB-Lite Slave Interface

**Figure 2:**
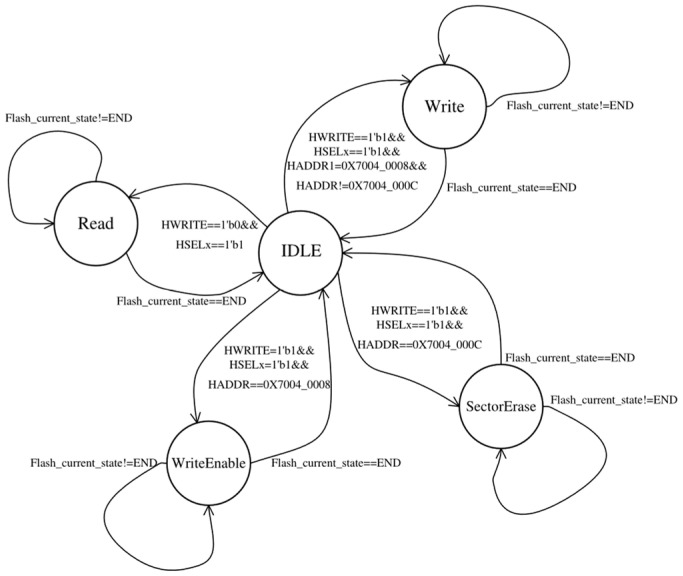
The finite state machine is implemented in the AHB-lite slave interface.

The AHB-lite slave interface functions as a medium that is responsible for real-time retrieval and parsing of read-and-write operation information from the AHB-lite bus while transferring the operations and related data to the data transceiver. Additionally, the current status or data of SPI-Flash also needs to be returned to the AHB-lite bus via the interface. The aforementioned medium functions are implemented through the utilization of a finite state machine, as depicted in Figure 2.

During the *IDLE* state, *HREADY* is pulled low upon the detection of control information pertaining to SPI-Flash on the AHB-lite bus. In the case where *HWRITE* is high, SPI-Flash control signals related to the *WriteEnable* or *SectorErase* operations will be generated in the *WriteEnable* or *SectorErase* state and output to the data transceiver when *HADDR* is detected to be equal to a functional address. Any other *HADDR* within the SPI-Flash memory space will be translated into a physical address in SPI-Flash by subtracting the base address 0x7000_0000, which will then be considered as the target write address. The *HWDATA* of the next cycle will be output to the data transceiver as the expected target word to be written. Specifically, if 0x7004_000C is detected, then the target erase sector address will be converted from *HWDATA* of the next cycle. Similarly, if *HWRITE* is low and *HADDR* falls within the SPI-Flash address space, a *READ* request along with the target read address and size information, will be outputted to the data transceiver.

**Figure 3:**
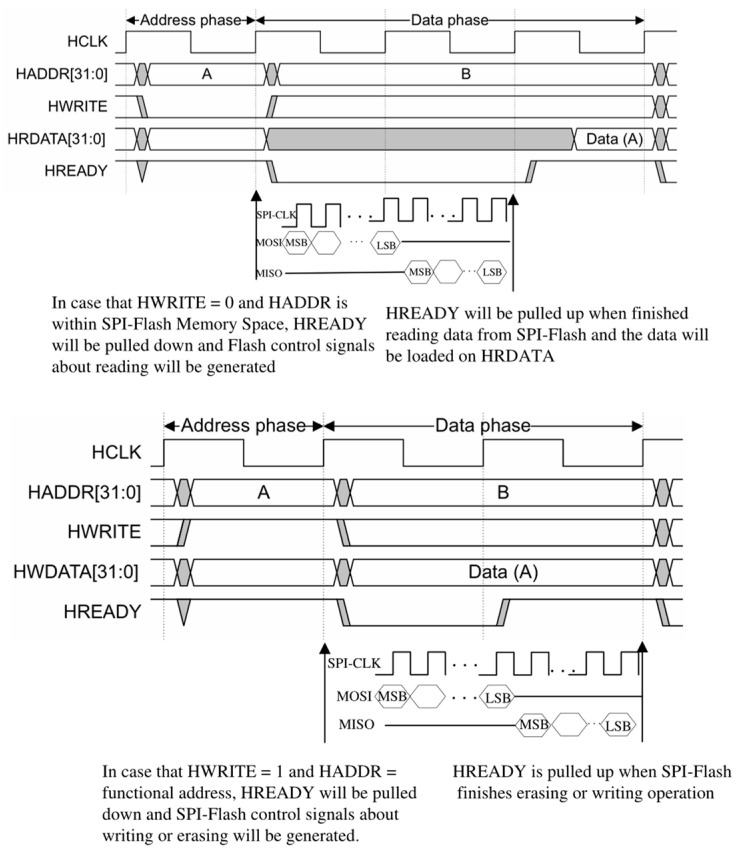
The HREADY signal control in AHB-lite slave interface.

The AHB-lite slave interface effectively manages SPI-Flash operations by dynamically adjusting *HREADY* based on the detection results from the AHB-lite slave interface and the current state of the SPI-Flash provided by the data transceiver, as illustrated in Figure 3. The mandatory approach compensates for the limitation of the embedded system where the SPI-Flash controller cannot utilize an interrupt mechanism to respond to the AHB-lite master before the completion of interrupt configuration in the boot phase.

### 2.3. The Design of the Data Transceiver

**Figure 4:**
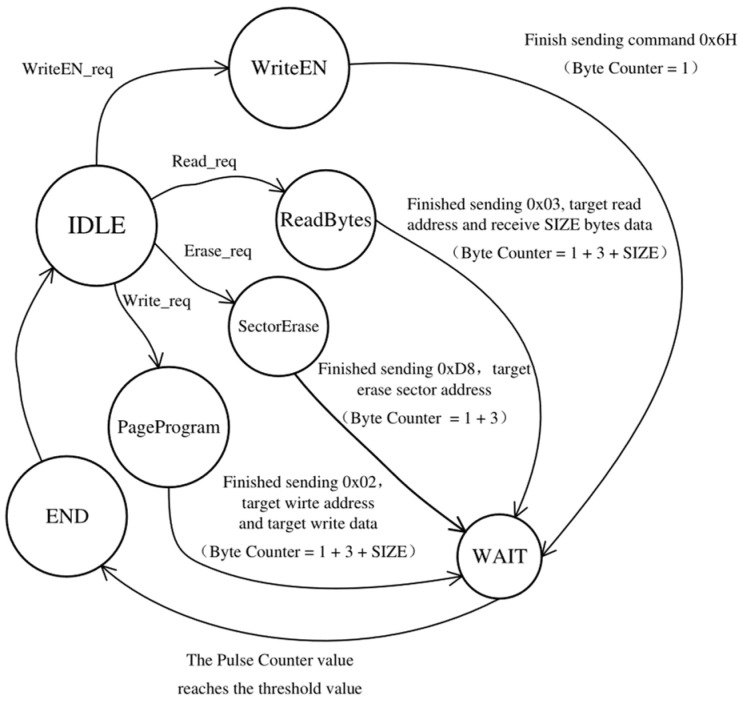
The finite state machine implemented in the data transceiver.

The data transceiver is responsible for controlling the serial–parallel converter to facilitate SPI transmission based on the SPI-Flash control signals received from the AHB-lite salve interface. Additionally, it combines bytes from the serial–parallel converter into data of the desired size and returns the data to the AHB-lite slave interface. Figure 4 depicts the finite state machine of the data transceiver.

**Figure 5:**
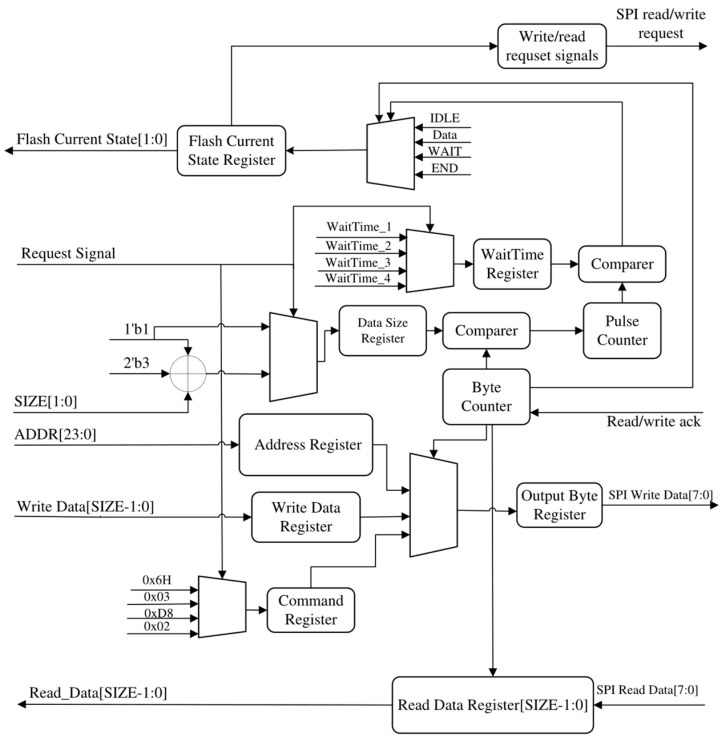
The structure of the data transceiver.

The SPI-Flash operations are executed in the *WriteEN*, *ReadBytes*, *SectorErase*, and *PageProgram* states upon receiving request signals from the AHB-lite slave interface in the *IDLE* state. Following data transfer, the SPI controller remains in the *WAIT* state until the pulse counter value reaches the threshold specified in the WaitTime register to ensure sufficient time is reserved for erasing or writing before executing the next operation. Each state can be detected by the AHB-lite slave interface for *HREADY* signal control. The finite state machine is implemented by the structure shown in Figure 5.

The data sent to the serial–parallel converter, the number of bytes to be sent and received, and the target address and the wait time for SPI-Flash operation are initialized into the SPI-Flash controller registers based on the SPI-Flash control signals from the AHB-lite slave interface. Table 2 illustrates the data initialized in the Command, Write Data, and Address registers for the *WriteEnable*, *SectorErase*, *Write*, and *READ* operations by taking Micron N25Q256A SPI-Flash as an example [15].

The byte counter is incremented by one when a read/write ack signal—indicating the successful transfer of one byte—is detected. Therefore, the progress of SPI transfer can be determined and controlled by utilizing the byte counter value as an index for retrieving each byte from the registers and concatenating the bytes from the serial–parallel converter. Meanwhile, SPI read/write request signals are output to the serial–parallel converter to determine whether data should be sent to or received from SPI-Flash. Subsequently, guided by the byte counter, the serial–parallel converter proceeds to output and receive each byte until its value matches that of the Data Size register. Finally, upon completion of the data transmission, a pulse counter is activated to wait for the SPI-Flash operations to conclude. Ultimately, based on both the values of the byte counter and pulse counter, the SPI-Flash current state is output in order to control the *HREADY* signal through the AHB-lite slave interface.

### 2.4. The Design of the Serial–Parallel Converter

**Figure 6:**
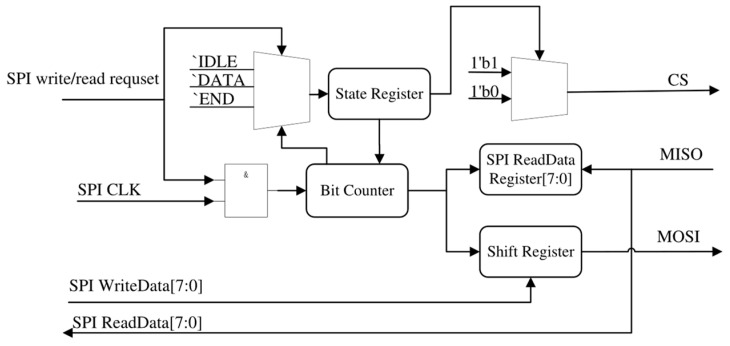
The serial–parallel converter structure.

The serial–parallel converter serves as the foundational layer of SPI communication directly with SPI-Flash, enabling it to transmit and receive 1 byte each time it receives an SPI write/read request signal. Its structure is shown in Figure 6.

The serial–parallel converter integrates a bit counter, similar to the data transceiver, which regulates the sending and concatenating of bits as well as *CS* (chip select). Upon detection of an SPI write or read request signal from the data transceiver, both *CS* and *SPI CLK* become active. Subsequently, on the rising edge of *SPI CLK*, the bit counter increments by 1 and functions as an index for placing each bit of *SPI WriteData[7:0]* onto the *MOSI* (Master out Slave in). Similarly, on the rising edge of *SPI CLK*, each bit from the *MISO* (Master in Slave out) is captured and stored in the SPI ReadData register using the bit counter value as an index. The bit counter is reset to 0, and CS is set to high when it reaches 7, indicating the completion of transmission in preparation for the next data transfer.

## 3. The Application of the Lightweight SPI-Flash Controller

The E906-SmartRun-Platform lacks support for non-volatile memory. Therefore, we have integrated the proposed lightweight SPI-Flash controller into the platform and designed the corresponding boot process. Moreover, the SPI-Flash controller functions as a temporary SPI-Flash programmer during the SoC prototype design process.

### 3.1. An Overview of the E906-SmartRun-Platform

The E906, developed by XuanTie company in China, is a high-performance and low-power RISC-V processor that employs a five-stage pipeline and an in-order single-issue mechanism. The XuanTie company offers an open-source project called E906-SmartRun-Platform, which serves as a SoC design and simulation platform based on the E906 processor. It includes RTL source code, a customized RISC-V toolchain, as well as relevant compilation and simulation scripts. However, no SPI-Flash controller is incorporated into this project. Therefore, it is necessary and feasible to integrate the lightweight SPI-Flash controller into the project.

The SoC developed in E906-SmartRun-Platform consists of three AHB-lite buses: the IAHB-lite bus for instructions, the DAHB-lite bus for data, and the SAHB-lite bus for peripherals. Access requests from the E906 processor are efficiently distributed to their respective bus interfaces based on the address mapping facilitated by the Bus Matrix Unit.

This platform facilitates the simulation of user program execution by directly initializing it into the IRAM, from which the E906 pipeline fetches instructions via the IAHB-lite bus during simulation. While this allows for program execution without SPI-Flash memory, it may not align with practical applications. Therefore, developing a boot process with the SPI-Flash controller would be meaningful for E906-SmartRun-Platform. Finally, the modified version of the SoC, enhanced with the boot process, was successfully ported to Genesys2 as a validation of the SPI-Flash controller. Refer to the integration manual [16] for more information about E906-SmartRun-Platform.

### 3.2. The Design of the Boot Process

The advantages of executing user programs in RAM over the XIP mode have been extensively discussed in the related work section above. Hence, it is imperative to develop a bootloader program and an arbiter to implement the boot functionality, which entails copying the user program from SPI-Flash to RAM and subsequently initiating the execution from RAM. These ideas were implemented by following the subsequent steps.

#### 3.2.1. VMA/LMA Space Allocation

The VMA/LMA [17] ranges of SPI-Flash and IRAM are presented in Table 3. According to the *E906-SmartRun-Platform Integration* manual, the start addresses of the SPI-Flash and IRAM are specified as 0x1000_0000 and 0x0001_0000, respectively, indicating that both the SoC’s start address in E906-SmartRun-Platform and the base address of the SPI-Flash controller should be configured as 0x1000_0000. Additionally, upon completion of program copying, the PC (program counter) should be set to 0x0001_0000.

#### 3.2.2. The Design of IAHB-Lite Bus Arbiter

**Figure 7:**
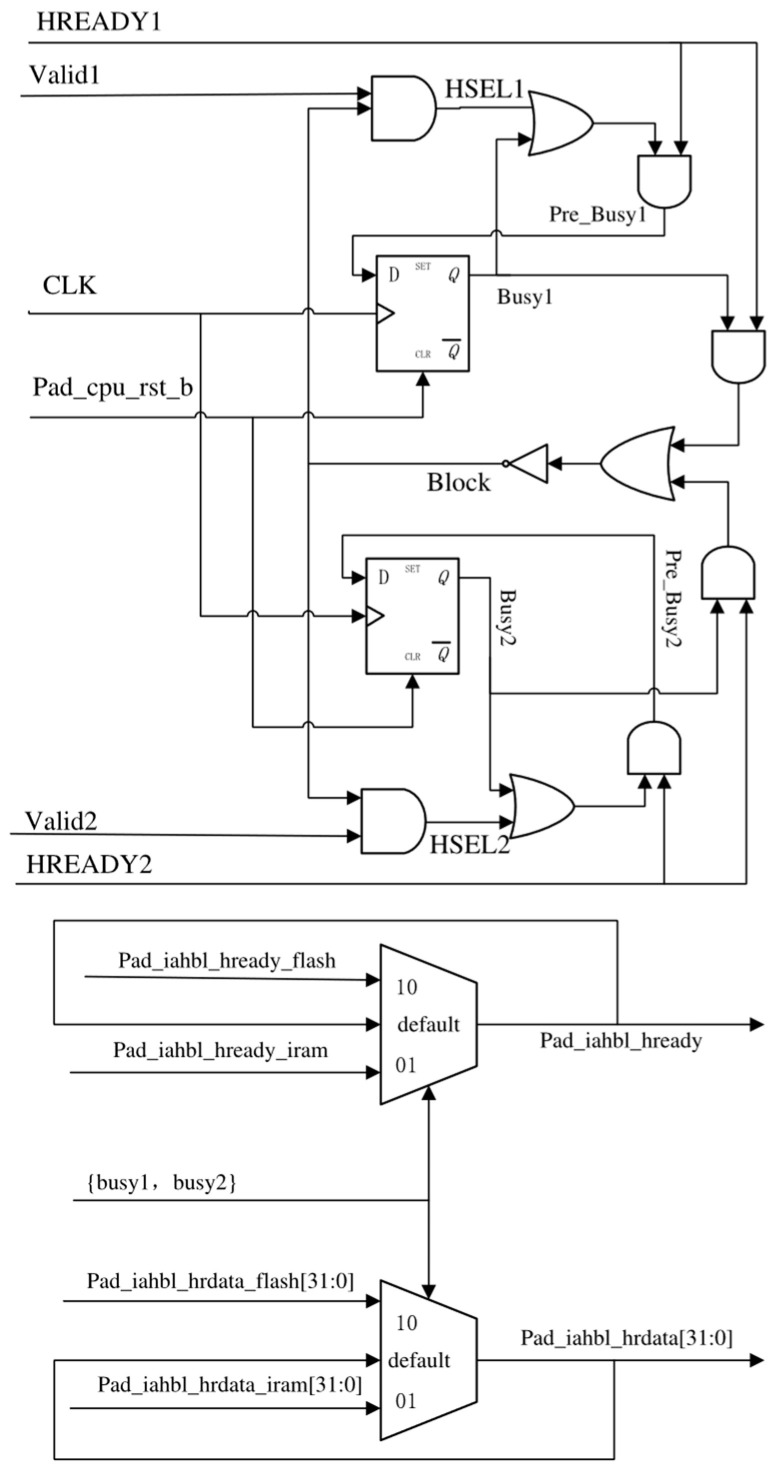
The IAHB-lite bus arbiter structure.

Both IRAM and SPI-Flash are slaves of the IAHB-lite bus; hence, it is essential to design an IAHB-lite bus arbiter to select the appropriate slave based on the *HADDR* signal value’s corresponding VMA/LMA space. The advantage of this SPI-Flash controller, which is directly connected to the AHB-lite bus, is manifested in the design process of the arbiter. The arbiter can be directly utilized as an upper layer for both SPI-Flash and IRAM, enabling the selection of either one during the bootloader program execution to carry out the read and write operations from the IAHB-lite bus. Conversely, if it were an APB interface controller, it would significantly complicate the design of the arbiter. The structure of the IAHB-lite bus arbiter is illustrated in Figure 7.

It should be noted that the suffix ‘x’ in the signal name denotes either ‘1’ or ‘2’. SPI-Flash and IRAM can be denoted by *slavex* when ‘x’ is 1 or 2. *Pre_busyx* is generated by a combinational logical circuit and determined by *HSELx* and *HREADYx* from the corresponding slave controller. *HSELx* = 1’b1 only if three conditions are met: (1) The address (*iahbl_pad_haddr*) falls within the VMA space of *slavex* (*Validx* = 1’b1); (2) the IAHB-lite transfer type is either SEQ or NONSEQ (*iahbl_pad_htrans[1]* = 1’b1), and there are no busy slaves (*block* = 0). When *Pre_busyx* equals 1, it indicates that *slavex* is about to start working or has already started working. Consequently, *slavex* will generate new output, which needs to be taken on the IAHB-lite bus. On the CLK rising edge, *busyx* is updated with the *pre_busyx* value. Therefore, when *busyx* = 1’b1, it signifies that *Pre_busyx* = 1’b1 in the previous cycle. The arbiter determines which slave’s output should be taken on the IAHB-lite bus based on their respective busy states ({*busy_1*, *busy_2*}).

#### 3.2.3. The Design of the Bootloader Program

The bootloader program mainly has two tasks to complete: copying the user program from SPI-Flash to IRAM and jumping to IRAM upon the completion of copying.

**Figure 8:**
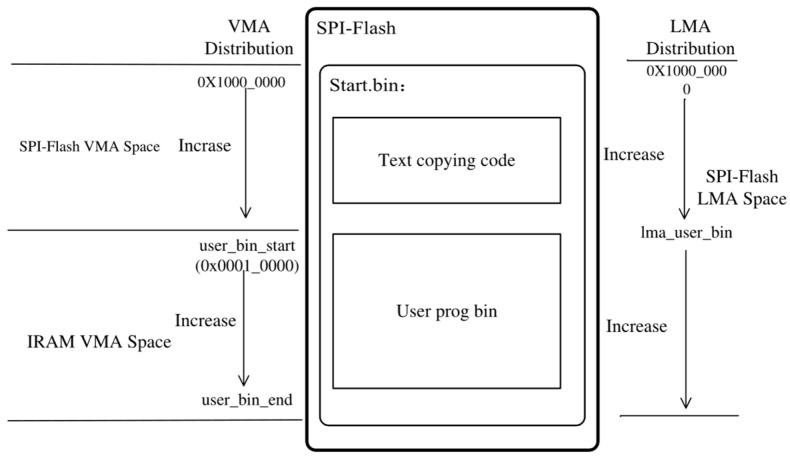
The VMA/LMA distribution of the bootloader program.

The absence of an operating system on bare metal necessitates the storage of the program expected to be downloaded in a dedicated section within the bootloader program. The VMA/LMA distribution of the bootloader program is shown in Figure 8.

The bootloader program consists of two parts: The first part contains the code responsible for copying the user program to IRAM, which is stored and executed in SPI-Flash, while the second part contains the user program bin expected to be copied. The VMA and LMA of the first part are located in SPI-Flash, as its code is stored and executed there. The VMA in the second part is assigned to the IRAM VMA space, serving as the destination address for data copying, while the LMA remains situated in the SPI-Flash LMA space, functioning as the source address during the copying process.

**Figure 9:**
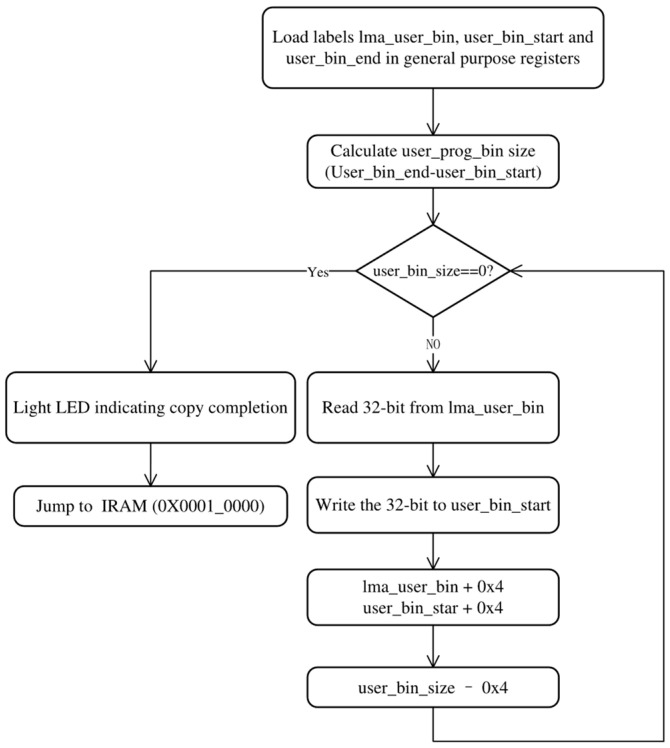
The flow chart of the bootloader program.

The first LMA and the initial and final VMAs of the user program binary are, respectively, labeled as “*lma_user_bin*”, “*user_bin_start*”, and “*user_bin_end*” in the linker script. These labels facilitate determining the source and destination addresses when writing bootloader programs in RISC-V assembly language. The flow of the bootloader program is shown in Figure 9.

The process of copying the user program from SPI-Flash to IRAM is executed through a loop, wherein the linker script’s provided labels are loaded and used to determine the source and destination addresses for the first copy as well as the size of the user program bin. During each iteration of this loop, a word (32-bit) is copied from IRAM to SPI-Flash while updating both *lma_user_bin* and *user_bin_start* + 0x4 in order to obtain new source and destination addresses. As such, it follows that by subtracting 0x4 from the original size of the user prog bin, we can accurately track its progress. After the completion of the copying process, the GPIO is manipulated to illuminate the LED indicator on Genesys2. Finally, the PC is set to 0x0001_0000 through the execution of RISC-V instructions LUI and JALR.

**Figure 10:**
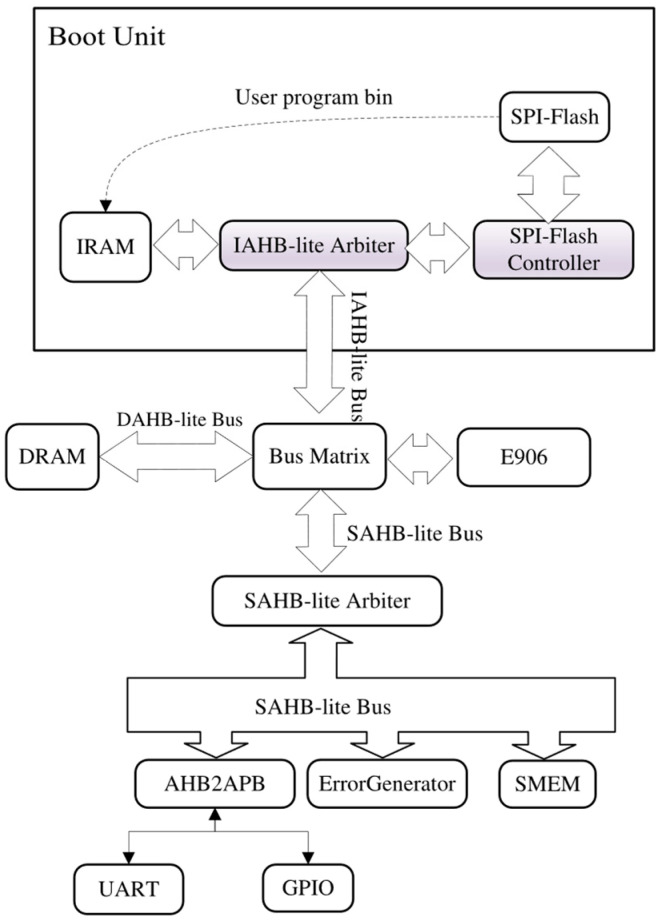
The structure of the SoC in the E906-SmartRun-Platform project enhanced by the boot unit.

The bootloader program was downloaded to the SPI-Flash using the method described in Section 3.3. Finally, the structure of the SoC in E906-SmartRun-Platform enhanced by a boot unit is depicted in Figure 10.

### 3.3. Downloading Bare-Metal Programs to SPI-Flash Using the Lightweight SPI-Flash Controller

**Figure 11:**
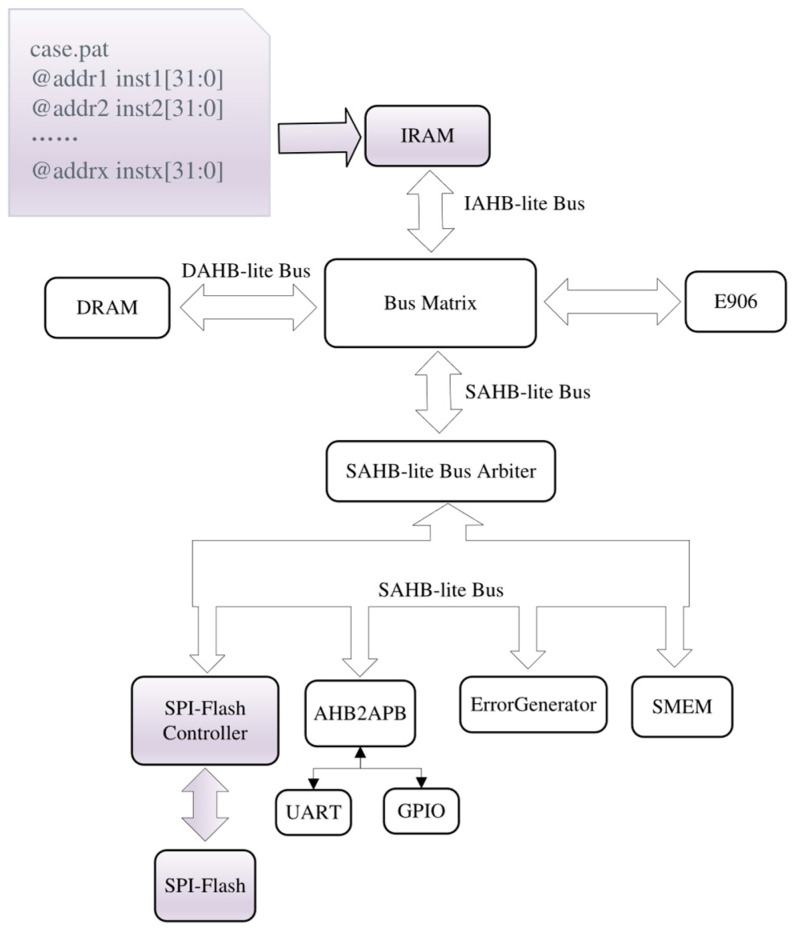
The structure of the SoC in E906-SmartRun-Platform in which the SPI-Flash controller can function as a bare-metal program downloader.

The proposed SPI-Flash controller can function as a program downloader during FPGA-based SoC prototype verification while executing a program called Prog2Flash based on the structure depicted in Figure 11. The Prog2Flash program is implemented using RISC-V assembly language, and the binary file of the user program expected to be downloaded to SPI-Flash is placed in a designated section of Prog2Flash. The Prog2Flash is converted into the Verilog readable file case.pat, facilitating the direct initialization of Prog2Flash to IRAM during the generation process of the bitstream for the structure depicted in Figure 11.

Therefore, downloading a program to SPI-Flash is essentially copying the program contained in Prog2Flash to SPI-Flash. The concept was implemented in E906-SmartRun-Platform through the subsequent five steps.

Step 1: Add the lightweight SPI-Flash controller as a slave of the SAHB-lite bus. The SPI-Flash controller can be integrated as a slave device on the SAHB-lite bus, following the same configuration process utilized for other peripheral controllers in the E906-SmartRun-Platform source code.

Step 2: Compile the program expected to be downloaded. In this step, programs written in C or RISC-V assembly language are converted into executable binary files (bin) using the customed RISC-V toolchain provided by XuanTie.

Step 3: Develop a linker script corresponding to Prog2Flash and compose Prog2Flash in RISC-V assembly language. Both Prog2Flash and its linker script can be created based on the bootloader program design described in Section 3.2, with the only requirement being the adjustment of the address distribution in the linker script and the removal of the jump function.

Step 4: It is necessary to build test programs that erase and read the SPI-Flash and output the results through UART to check whether the program has been downloaded successfully.

Step 5: Port the SoC in the E906 project to the Digilent Genesys2 board. Xilinx Vivado is used to generate bitstreams for the SoC structure, whose IRAM is loaded with some type of program. These bitstreams are then downloaded onto the Digilent Genesys2 board, where Prog2Flash and the programs built in Step 4 are executed.

### 3.4. The Test of Bare-Metal Program Download and System Booting Process

The simulation results and FPGA-based prototype verification demonstrate the successful completion of a program’s download and support for the boot function of the embedded system by the lightweight SPI-Flash controller.

**Figure 12:**
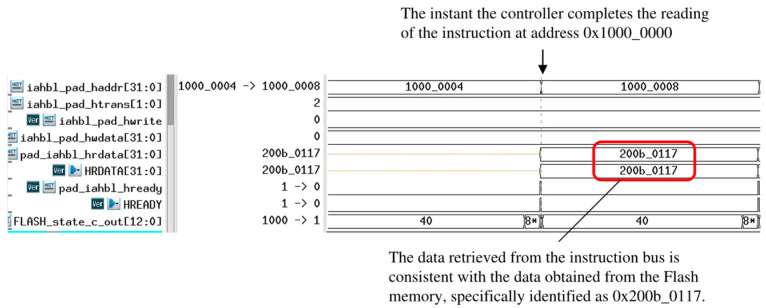
Fetching instructions from SPI-Flash.

**Figure 13:**
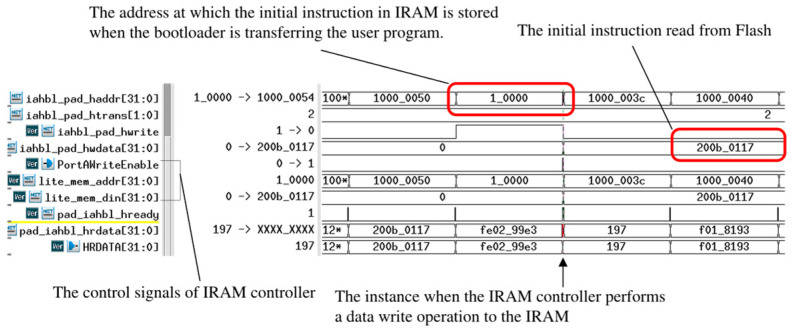
Copying the user program to IRAM from SPI-Flash.

**Figure 14:**
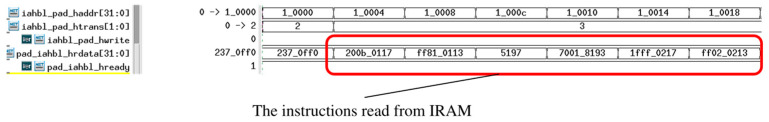
Jumping to IRAM and fetching instructions from IRAM.

During the boot process simulation conducted using VCS, the SPI-Flash content is replaced with a register array initialized by the boot loader program to ensure the instructions are returned to the IAHB-lite bus. Distinct stages of the boot process are depicted in Figure 12, Figure 13, and Figure 14, respectively.

It is clear that the *pad_iahbl_hready* signal remains consistent with the lightweight SPI-Flash controller under the influence of the IAHB-lite bus arbiter. The *HREADY* signal output by the lightweight SPI-Flash controller is pulled up at the instant the SPI-Flash controller completes the reading of the instruction (0x200b_0117) at address 0x1000_0000. The instruction is then loaded onto *pad_iahbl_hrdata*, providing input to the processor.

The control information regarding the writing of data to IRAM remains unchanged until the lightweight SPI-Flash controller completes the last read operation. Subsequently, the IRAM controller is selected (*lite_mmc_hsel* = 1’b1), and its signals indicate that the instruction at the first address of the user program is written to 0x0001_0000, which falls within the IRAM VMA space (*lite_mmc_addr* = 0x0001_0000, *lite_mmc_din* = 0x200b_0117, *PortWriteEnable* = 1’b1). Meanwhile, *pad_iahbl_hready* remains consistent with the IRAM controller under the influence of the IAHB-lite bus arbiter.

**Figure 15:**
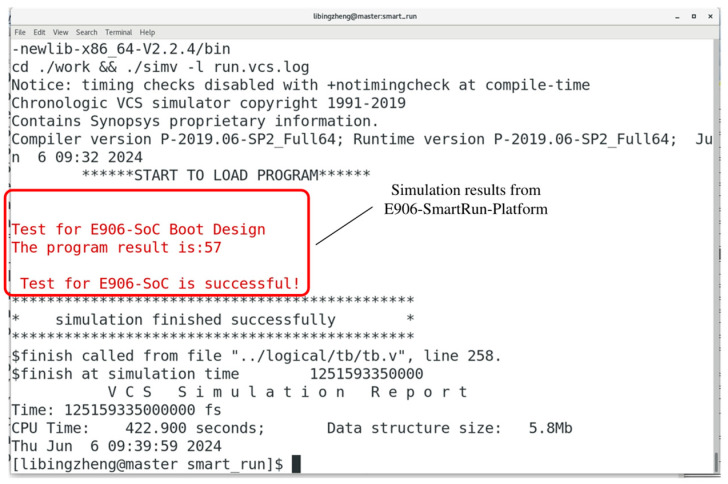
The simulation results of the boot process designed for the E906-SmartRun-Platform project based on the proposed SPI-Flash controller.

The instructions are fetched from IRAM after the jump, as indicated by the IAHB-lite bus signal *iahbl_pad_haddr* falling within the IRAM VMA space. Furthermore, the fetched instructions from IRAM align consistently with the disassembly results. The final running result of the program is output to the Linux terminal under the simulation incentive provided by the E906-SmartRun-Platform project, as depicted in Figure 15. This outcome aligns with expectations and confirms the successful completion of the simulation.

The porting of the E906-SmartRun-Platform project to FPGA, complemented by the boot process based on the proposed SPI-Flash controller, is crucial for error identification beyond simulation capabilities. The porting procedure utilizes the Digilent Genesys2 development board, which is equipped with the Xilinx Kintex-7 FPGA chip, and the Pmod SF3 module, which features Micron MT25QL256ABA. Firstly, the SoC architecture illustrated in Figure 11 is synthesized using Xilinx Vivado 2021.2. Subsequently, the generated bitstream is downloaded onto the Gensys2 platform after inserting Pmod SF3 into the Pmod interface of Genesys2. Upon executing Prog2Flash initialized to IRAM, the LED indicator illuminates, indicating a successful download of the test program to Pmod SF3. Following this step, the bitstream of the SoC structure shown in Figure 10 is programmed onto Genesys2, initiating execution of the test program stored in Pmod SF3.

**Figure 16:**
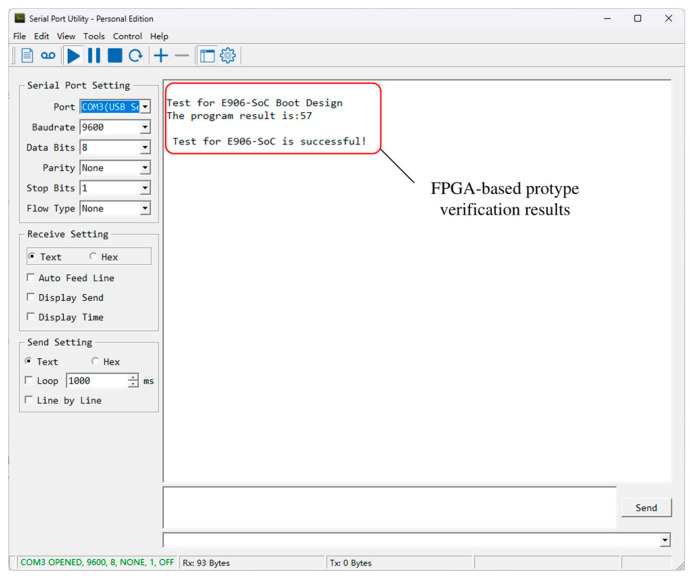
The FPGA test results of the boot process designed for the E906-SmartRun-Platform project based on the proposed SPI-Flash controller.

The synthesis results of the SoC based on the E906-SmartRun-Platform project and the proposed SPI-Flash controller under Xlinx Vivado 2021.2 are shown in Table 4. As the printf() functions within the user program are redirected to UART, the final program execution results are output to a serial debugging assistant, as depicted in Figure 16.

The perfect alignment between the simulation results and FPGA test results, as depicted in Figure 15 and Figure 16, further validates the successful implementation of the proposed SPI-Flash controller and its application in E906-SmartRun-Platform. The results presented in Table 4 also demonstrate that the proposed SPI-Flash controller exhibits a significantly reduced utilization of FPGA resources. With the exception of IO, each resource only occupies a negligible proportion of E906-SmartRun-Platform. The synthesis results indicate that the SPI-Flash controller is equipped with up to 76 IOs as a result of the wide bit width requirments of the AHB-lite bus. However, when integrated into the SoC, only three IO ports, namely CS, MISO, and MOSI, are truly necessary.

Because the interface of the SPI-Flash controller is designed based on the standard AHB-lite bus interface, any embedded system equipped with an AHB-lite bus structure can integrate the SPI-Flash controller. Additionally, from the integration of the SPI-Flash controller in the SoC of the E906-SmartRun-Platform project, it can be seen that the SPI-Flash controller has strong portability. This is because the operation of SPI-Flash is based on the mode of memory mapping; that is, it only needs to read and write the corresponding address through assembly or C language, and the AHB-lite slave interface of the SPI-Flash controller can decode the address to complete the corresponding operation on SPI-Flash. In other words, the process of integrating the SPI-Flash controller on different SoCs is similar; only the bootloader and its linker scripts need to be adjusted according to the actual situation. In addition, the SPI-Flash controller is lightweight, has a small area, and has low power consumption, so it will not cause too much of a burden on the SoC.

## 4. Performance Analysis

The SPI-Flash controller is synthesized and constrained using a design compiler (DC) based on the SMIC 180 nm process, achieving a maximum operating frequency of 125 MHz, which satisfies the requirements for an embedded system boot process design. The area measures 19,861 units, with a power consumption of 1.2031 mW. These results align with our lightweight design objective.

By referring to the data sheet of the SPI-Flash Micron N25Q256A used in the experiment, we calculated that driven by the maximum operating frequency (125 MHz) clock of the SPI-Flash controller, the maximum data throughput ($B_{max}$) of the lightweight SPI-Flash controller can reach 13.62 MB/s. However, the official data sheet states that the maximum operating frequency of N25Q256A under the SPI protocol is about 108 MHz, and the maximum data throughput that N25Q256A can achieve with the Quad-SPI protocol is about 54 MB/s. Therefore, the maximum data throughput of N25Q256A under the SPI protocol is about 11.77 MB/s. Even according to the rate relationship between the Quad-SPI protocol and SPI protocol, the maximum data throughput that can be achieved is about 13.5 MB/s. Therefore, the SPI-Flash controller can make full use of the communication rate of N25Q256A under the SPI protocol and still has good performance under the premise of realizing a lightweight design.

(1) $$B_{max}=\frac{64*f_{max}}{70}\approx 13.62\;MB/s$$

(The number 64 in Equation (1) indicates that a total of 64 bits of data are transmitted throughout the entire operation, encompassing 8 bits for the commands, 24 bits for address lines, and 32 bits for data).

The proposed controller exhibits lightweight and efficient characteristics, as evidenced by the comparative outcomes between the proposed controller and other designs in the relevant literature presented in Table 5. It is noteworthy that the maximum frequency of the SPI-Flash controller reaches 5/8 of the maximum frequency of the 200 MHz listed in the table. Meanwhile, its area and power consumption are reduced by at least half compared to other references. Unfortunately, due to the power consumption and area constraints, this SPI-Flash controller lacks hardware support for enhancing data throughput, thereby resulting in suboptimal performance in terms of data throughput. However, our experimental findings demonstrate that it satisfactorily meets the startup requirements of small RAM-embedded systems.

## 5. Conclusions

We have developed a lightweight SPI-Flash controller equipped with AHB-lite buses. The SPI-Flash controller can be utilized to facilitate the development of a boot process supporting RAM-based program execution and serve as a temporary SPI-Flash downloader during FPGA-based SoC prototype verification. Additionally, we have successfully implemented a boot process for the open-source SoC project E906-SmartRun-Platform based on the SPI-Flash controller, enabling efficient copying of user programs from SPI-Flash to RAM during startup. The simulation and FPGA test results demonstrate remarkable performance during the boot process and program downloading.

The source code has been uploaded to the GitHub repository.

However, the lightweight design of the controller comes at the expense of simplifying the boot process and sacrificing extra SPI-Flash operations. As a result, the applicability of this controller may be limited in scenarios involving more complex SPI-Flash operations. Therefore, it is essential to implement additional enhancement measures in future endeavors.

## Figures and Tables

**Table 1:** Space allocated for the controller (Base Address = 0x7000_0000).

Address	Offset
WriteEnable	0x0004_0008
SectorErase	0x0004_000C
Write/Read	[0: 0x0004_0000]
Memory Address	[0: 0x0004_0000]

**Table 2:** Data transceiver registers’ initialization for SPI-Flash operations.

Operations	Command	Address	WriteData	DataSize
WriteEnable	0x6H	NULL	NULL	1
SectorErase	0xD8	SectorADDR[23:0]	NULL	1 + 3
Write	0x02	WriteADDR[23:0]	WriteData[31:0]	1 + 3 + 4
Read	0x03	ReadADDR[23:0]	NULL	4 + ReaSize

**Table 3:** VMA/LMA space range allocated for SPI-Flash and IRAM.

VMA/LMA Space	Start	End
SPI-Flash	0x1000_0000	0x1000_0000 + 256 KB
IRAM	0x0001_0000	0x0001_0000 + 256 KB

**Table 4:** The FPGA synthesis results of the proposed SPI-Flash controller and the SoC platform.

	LUT	LUTRAM	FF	BRAM	IO	BUFG	PLL
SPI-Flash Controller	107	-	138	-	76	2	-
SoC Platform	33,652	464	14,639	384	25	9	1

**Table 5:** The comparative outcomes between the proposed controller and other designs in the relevant literature.

Literature	Manufacturing Technology	Control Bus	SDA Lines	Area (µm^2^)	Frequency (MHz)	Power (mW)
[18]	SMIC 55 nm	AHB	8	62,758	200	3.44
[19]	HuaHong 110 nm	-	4	68,020	200	3.76
[20]	65 nm	-	4	567,096	70	3.83
[21]	SMIC 180 nm	APB	4	-	200	8.47
[22]	SMIC 180 nm	AHB	-	1,252,143	121	-
This Work	SMIC 180 nm	AHB-lite	2	19,861	125	1.2031

## Data Availability

The data presented in this study are available upon request from the corresponding author.
